# Supplementary figures and images for: Proteomics Profiling with SWATH-MS Quantitative Analysis of Changes in the Human Brain with HIV Infection Reveals a Differential Impact on the Frontal and Temporal Lobes
Source: Brain Sci. 2021 Oct 28;11(11):1438. doi: 10.3390/brainsci11111438 (PMC8615382; doi:10.3390/brainsci11111438)

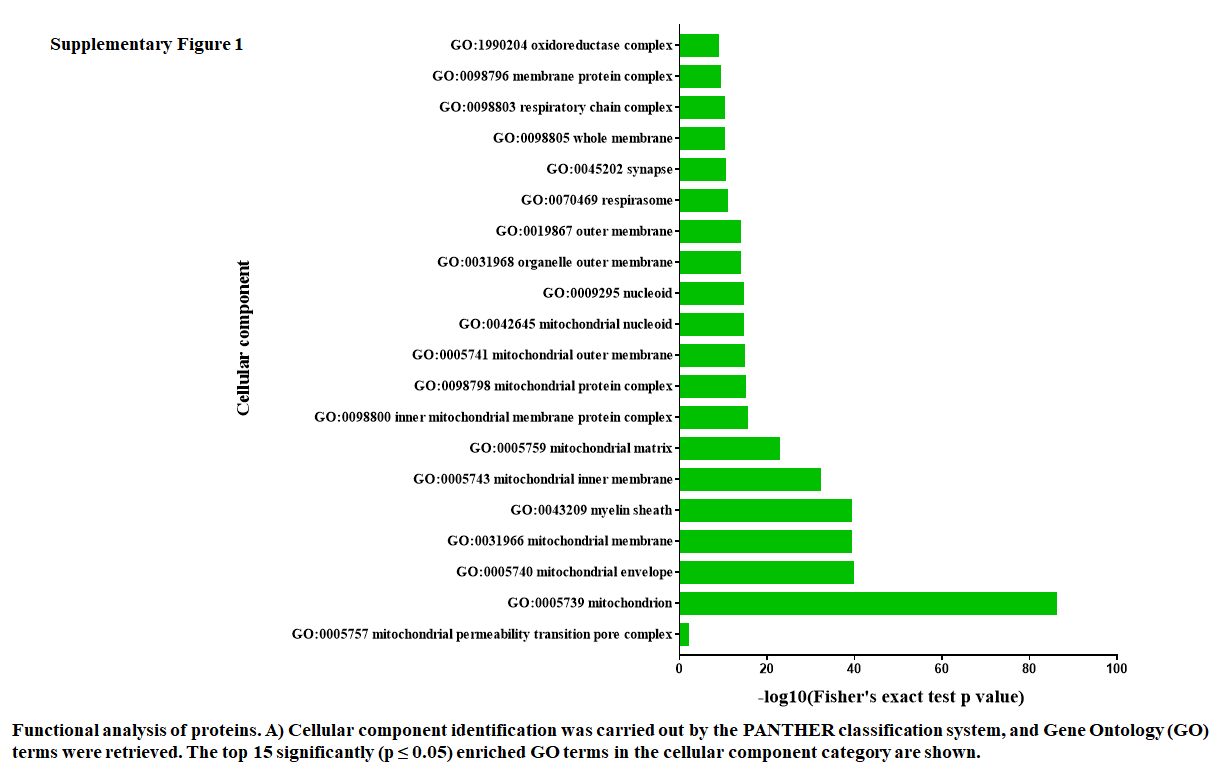

Supplement: Supplementary file 1 [file brainsci-11-01438-s001.zip › Supplementary Figure 1.tif]

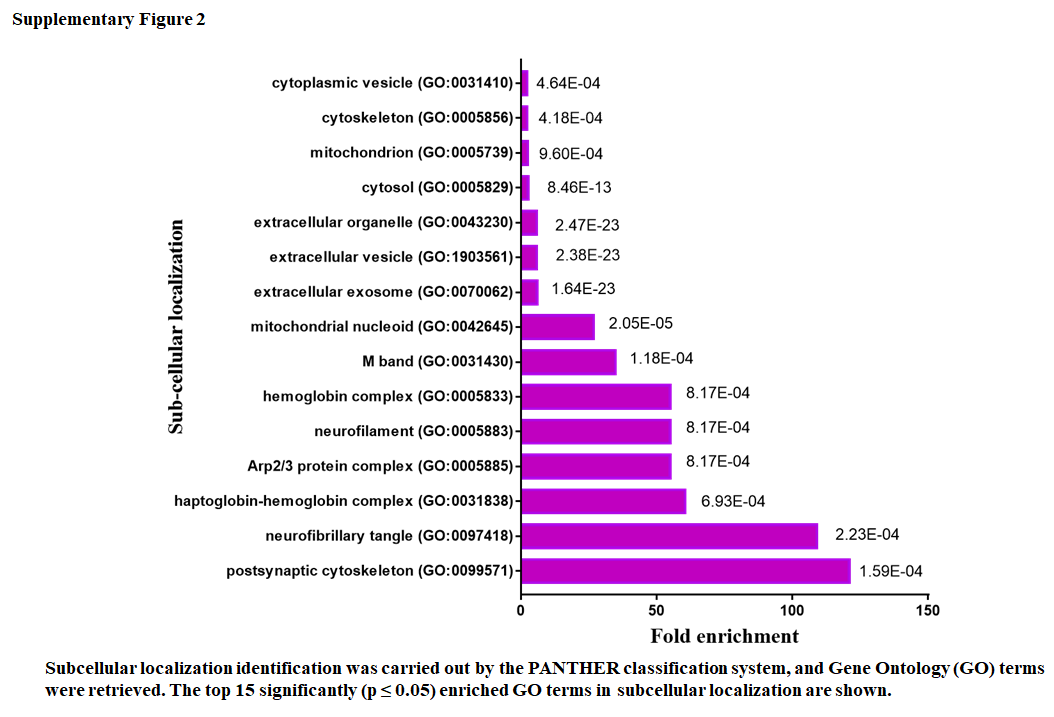

Supplement: Supplementary file 1 [file brainsci-11-01438-s001.zip › Supplementary Figure 2.tif]
